# Label-free as-grown double wall carbon nanotubes bundles for *Salmonella typhimurium* immunoassay

**DOI:** 10.1186/1752-153X-7-102

**Published:** 2013-06-14

**Authors:** Niramol Punbusayakul, Saikat Talapatra, Pulickel M Ajayan, Werasak Surareungchai

**Affiliations:** 1 School of Agro-Industry, Mae Fah Luang University, Chiang Rai 57100, Thailand; 2 Department of Physics, Southern Illinois University Carbondale, Carbondale, IL 62901, USA; 3 Department of Mechanical Engineering & Materials Science, Rice University, Houston, TX 7705, USA; 4 School of Bioresources and Technology, King Mongkut’s University of Technology Thonburi, Bangkok 10140, Thailand

## Abstract

**Background:**

A label-free immunosensor from as-grown double wall carbon nanotubes (DW) bundles was developed for detecting *Salmonella typhimurium*. The immunosensor was fabricated by using the as-grown DW bundles as an electrode material with an anti-*Salmonella* impregnated on the surface. The immunosensor was electrochemically characterized by cyclic voltammetry. The working potential (100, 200, 300 and 400 mV *vs.* Ag/AgCl) and the anti-*Salmonella* concentration (10, 25, 50, 75, and 100 μg/mL) at the electrode were subsequently optimized. Then, chronoamperometry was used with the optimum potential of 100 mV *vs.* Ag/AgCl) and the optimum impregnated anti-*Salmonella* of 10 μg/mL to detect *S. typhimurium* cells (0-10^9^ CFU/mL).

**Results:**

The DW immunosensor exhibited a detection range of 10^2^ to 10^7^ CFU/mL for the bacteria with a limit of detection of 8.9 CFU/mL according to the IUPAC recommendation. The electrode also showed specificity to *S. typhimurium* but no current response to *Escherichia coli*.

**Conclusions:**

These findings suggest that the use of a label-free DW immunosensor is promising for detecting *S. typhimurium*.

## Background

*Salmonella typhimurium* is among the most dangerous bacteria reported by the Centers for Disease Control and Prevention (CDC). It is involved in various types of disease outbreaks. The foods most commonly linked to the outbreaks include ground beef, African dwarf frog, raw milk and cheese, peanut butter and tomatoes [1-4]. The conventional agar method is usually used for detecting the bacteria, but it is time consuming. A rapid method is needed for food industries to determine the presence of *Salmonella* to ensure food safety. Immunoassay is one of the rapid methods used for detecting *Salmonella* and has long been used for the detection of *S. Typhimurium*; however, enzymes or nanoparticle labelling with prior enriched cultivation is regularly required to make detection possible [5-8].

Carbon nanotubes (CNTs) have become increasingly interesting for fabricating electrodes for immunoassay. In addition to CNTs’ capability for promoting electrochemical reactivity of many types of biomolecules, the CNTs have high aspect ratio compared to other types of nanomaterials. This makes them compatible with a wider variety of biological species such as enzymes, protein, and so on. [9,10]. Such biological species are known to be able to enhance the CNTs’ electron transfer, making the CNTs a promising electrode material. However, some have reported that the architecture of the CNTs could affect electrode behaviour [11,12]. Recently, various CNTs architectures have been producing and their characterization for immunoassay has not yet been reported. Our previous works have investigated the use of different CNT macroarchitectures, including MWCNT and SWCNT mat, vertically aligned MWCNT, DWCNT fibre [13,14]. The thread-like and the mat of as-grown SWCNT and DWCNT, with glassy carbon electrode properties, have been compared and investigated and the results indicated DWCNT to be a superior electrode material for electrochemistry over others [15]. Previous works also confirm DWCNT properties to have fast electron transfer with significant overpotential reduction as well as wide working potential for various species [16,17]. In this work, the first use of a thread-like, as-grown DWCNT for fabricating a label-free immunosensor is reported. This is to provide a new architecture for the fascinating CNTs with label-free immunosensor in order to provide high sensitivity electrode for *S. typhimurium* detection.

## Results and discussion

### Electrochemical characterization

**Figure 1:**
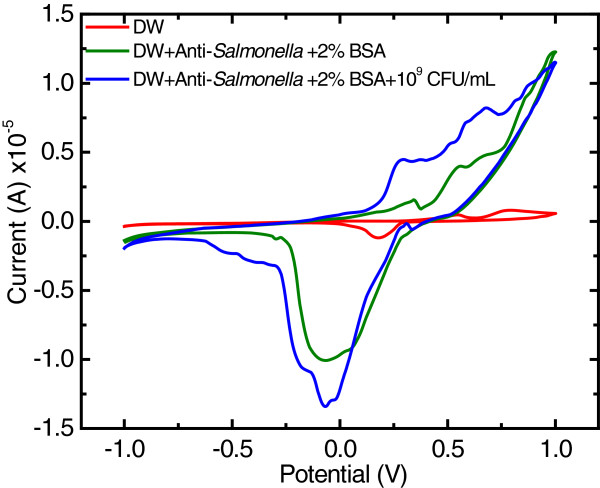
**Cyclic voltammograms at the (red) DW electrode, (green) MAb-DW electrode, and (blue) MAb-DW electrode with immobilized cells when the potential was cycling from −1.0 to 1.0 V *vs. *Ag/AgCl at a scan rate of 20 mV/sec in citrate phosphate buffer (0.05 M, pH 5.5) solution.**

The results showed that both the oxidation and reduction peaks at the electrodes were clearly observed after the electrode was modified with anti-*Salmonella* antibody and the microbial cells (Figure 1). However, it was found that the ration of the oxidation peak current at the cells-incorporated-MAb-DW electrode to the peak current at the MAb-DW electrode (Iox at the cells-MAb-DW electrode/Iox at the bare MAB-DW electrode) was higher (3.75) than the ration of the reduction current response at the cells-incorporated-MAb-DW electrode to the background current at the MAb-DW electrode (1.30). This may be due to the typical negative-charged cell wall of the gram-negative bacteria, which contains lipopolysaccharide, and maybe donated electrons to the electrode. Therefore, the oxidation potential was applied for further immunoassay optimization by using chronoamperometry. The increased current response indicated the incorporation of the protein, the MAb and the microbial cells, at the DW electrode. This suggests a high sensitivity of the electrode for protein recognition even though no mediator is incorporated, which also implies the label free capability of the electrode material for protein detection.

### Working potential optimization for immunoassay

**Figure 2:**
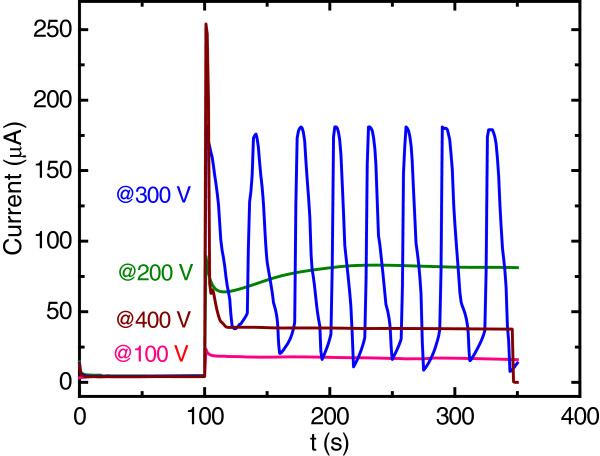
**Chronoamperograms at the 10 μg/mL MAb incorporated-DW electrode with the applied working potential of (pink) 100 V, (green) 200 V, (blue) 300 V, and (brown) 400 V *vs. *Ag/AgCl without the cell incorporation in citrate phosphate buffer (0.05 M, pH 5.5) solution.**

The working potential at the MAb-DW electrode was optimized at a fixed concentration of MAb of 10 μg/mL in the working solution with citrate-phosphate buffer (0.05 M, pH 5.5) and without the cells incorporated. The working potential applied at the electrode was varied to 100, 200, 300 and 400 mV according to the CV measurements. The results showed that all applied working potentials provided well defined chronoamperograms, except for 300 V (Figure 2). However, a distorted signal was obtained when the working potential of 400 V was applied.

It was also found that the background current at the electrode increased with the increasing working potential; however, it dropped at the working potential of 400 V. In addition to providing the lowest background current, the working potential of 100 V also exhibited a consistent current response throughout the measurement time compared to the others (Figure 2). The lower background current response is normally known to provide better sensitivity, so the working potential of 100 mV was then chosen to further optimize the MAb concentration, to be immobilized onto the electrode for an immunoassay.

### MAb concentration optimization for immunoassay

**Figure 3:**
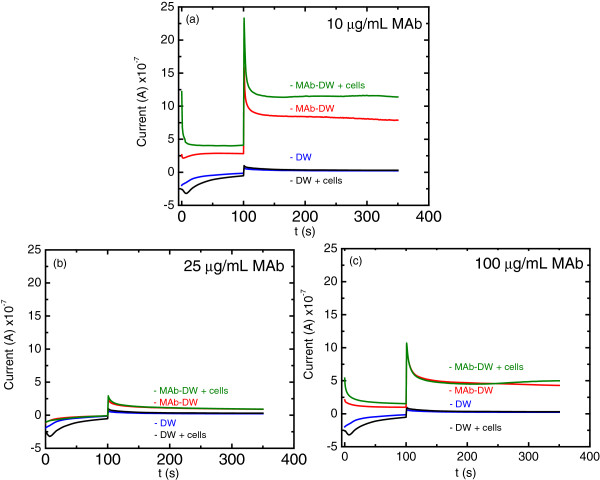
**Chronoamperograms at (blue) the bare DW electrode, (black) the bared DW electrode with cells, (red) the MAb-DW electrode, and (green) the MAb-DW electrode with the cell incorporation (10**^**9**^ **CFU/mL) at the MAb concentration of (a) 10 μg/mL, (b) 25 μg/mL, and (c) 100 μg/mL at the working potential of 100 V *vs. *Ag/AgCl in citrate phosphate buffer (0.05 M, pH 5.5) solution.**

The potential of 100 V *vs.* Ag/AgCl was applied for MAg concentration optimization onto the DW electrode in order to detect *S. typhimurium* cells. Figure 3 shows the chromatograms obtained when different MAb concentrations were applied onto the DW electrode. It was found that the bare DW electrode with and without the incorporated cells after BSA blocking exhibited no difference in current response when there was no MAb immobilized on the electrode. This confirms that there is no non-specific adsorption of the cells on the electrode without MAb immobilization. In addition, the signal at the bare electrode was lower than the signal obtained at the MAb-DW electrode at all ranges of MAb concentrations. These results are consistent with those obtained from CV characterization, which confirms the protein incorporation process onto the electrode surface.

From Figure 3a, the signal was clearly observed with a well-defined chronoamperogram when the cells were attached at the electrode containing 10 μg/mL of MAb. However, there was a negligible difference of the current response and in the background current observed when the higher MAb concentrations were immobilized onto the electrode surface, as shown in Figure 3b and 3c. Furthermore, the current response at the electrode containing 100 μg/mL MAb exhibited overlapping chromatogram to the background current. This indicates that the MAb at the concentration of 100 μg/mL might be the excess concentration that could limit electrode sensing capability. These results, therefore, suggest that the MAb at the concentration of 10 μg/mL is the optimal concentration for immunoassay, and it will be further used for testing the sensitivity of the electrode for the detection of *Salmonella* cells.

### Immunoassay at the MAb-DW electrode

**Figure 4:**
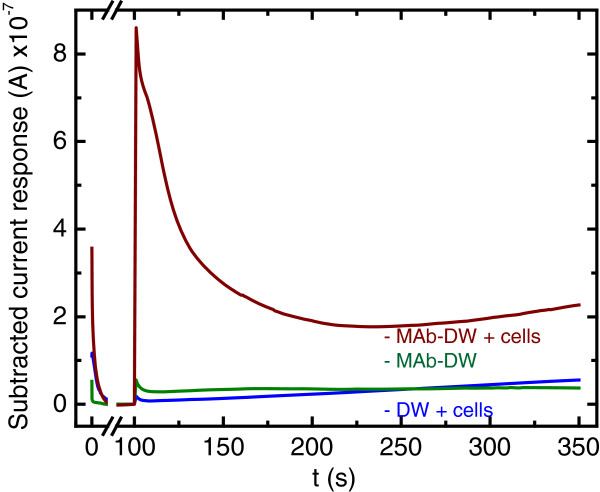
**Chronoamperograms at (blue) the bare DW electrode, (green) the DW electrode with MAb at the concentration of 10 μg/mL, and (red) the MAb-DW electrode with cells (10**^**9**^ **CFU/mL) at the working potential of 100 V *vs. *Ag/AgCl in citrate phosphate buffer (0.05 M, pH 5.5) solution.**

The MAb at the concentration of 10 μg/mL and the working potential of 100 mV *vs.* Ag/AgCl was used for *Salmonella typhimurium* detection. It was found that there was no signal of the cells obtained when only the blocking agent was immobilized onto the electrode surface, while the signal of the cells was obviously observed at the MAb-DW electrode (Figure 4). This confirms that the electrode is not able to detect any cells when MAb is not immobilized onto the electrode surface.

**Figure 5:**
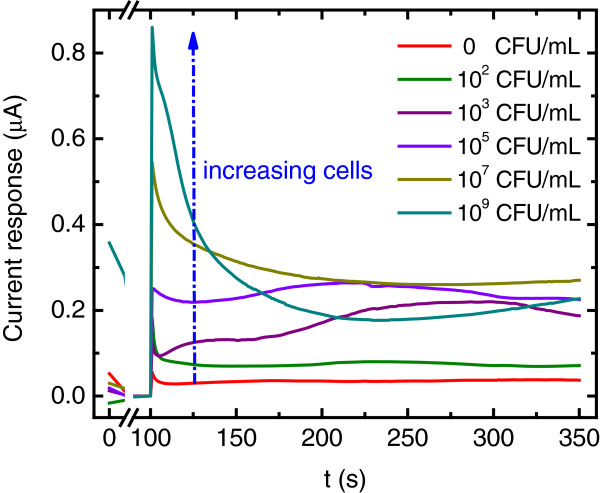
**Chronoamperograms at the MAb-DW electrode with 10 μg/mL MAb at the working potential of 100 V *vs. *Ag/AgCl in citrate phosphate buffer (0.05 M, pH 5.5) solution with various cell concentrations (0 to 10**^**9**^ **CFU/mL).**

**Figure 6:**
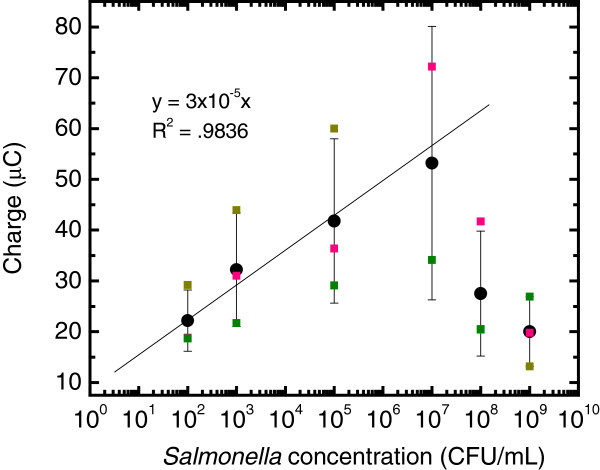
**A plot of *Salmonella typhimurium cell *(CFU/mL) *vs. *the subtracted charge (μC) obtained at the MAb-DW electrode.**

Figure 5 shows chromatograms obtained at the MAb-DW electrode with different cell concentrations (10-10^9^ CFU/mL) in a citrate phosphate buffer (0.05 M, pH 5.5) solution. No signal was observed when the cell concentration was lower than 10^2^ CFU/mL. In addition, it was found that the obtained current responses at the MAb-DW electrode increased with the increasing concentration of cells. A linear relationship was observed when the charge after the signal subtraction was plotted versus the log of the cell concentration at the range of 10^2^ to 10^7^ CFU/mL with R^2^ = 0.9836, as shown in Figure 6. The plot shows that the charge increased with the increasing of cell concentration until it reached 10^7^ CFU/mL. At this point, the charge was observed to decline. The results were similar to when the cell concentration was plotted against the subtracted signal (not shown). This indicates that the detection range at the MAb-DW electrode is limited to the S. typhimurium cells that ranged from 10^2^ to 10^7^ CFU/mL. The limit of detection calculated according to the IUPAC recommendation was 8.9 CFU/mL.

**Figure 7:**
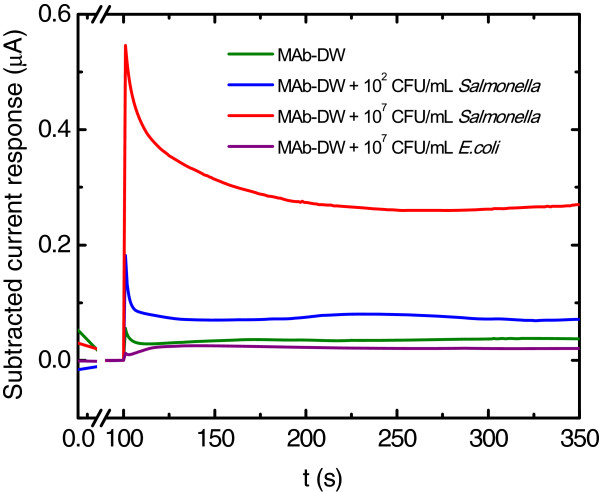
**Chronoamperograms at the MAb-DW electrode with 10 μg/mL MAb at the working potential of 100 V *vs. *Ag/AgCl in citrate phosphate buffer (0.05 M, pH 5.5) solution with *Salmonella typhimurium *cells at (blue) 10**^**2**^ **CFU/mL, (red) 10**^**9**^ **CFU/mL, and (purple) with *Escherichia coli *at 10**^**7**^ **CFU/mL.**

The electrode was also tested for non-specific sensing to another potent foodborne pathogen, *Escherichia coli* (Figure 7). The results showed that the electrode was not able to detect *E. coli* cells even when the cell concentration was as high as 10^7^ CFU/mL, whereas the same electrode was able to detect the *S. typhimurium* cells at the concentrations of 10^2^ and 10^7^ CFU/mL. These results emphasize the specificity of the electrode capability to detect *S. typhimurium*. Table 1 shows that the label-free DW electrode is promising for immunosensor fabrication by providing low LOD for *S. typhimurium* detection. In addition to the lower LOD, the fabrication ease of the platform also poses a challenge for further developing this novel material for detecting other microorganisms. However, other techniques and strategies could be combined in order to enhance the sensitivity of the electrode.

**Table 1:** **Immunosensors used for *Salmonella spp. *determination**

**Platform**	**Technique**	**Detection range (CFU/mL)**	**LOD**	**Ref.**
Ab-Ab/Au/Cu (SW)	ASV	1.30 × 10^2^ - 2.6 × 10^3^	98.9	[19]
MgB/Ab-Ab/AP (SW)	Colorimetry	-	8 × 10^3^	[20]
MgB/Ab-Ab/AP (SW)	Colorimetry	2.2 × 10^4^-2.2 × 10^6^	2.2 × 10^4^	[21]
Ab (LB)	AWD	1.8 × 10^6^ to 10^9^	-	[22]
MET-Ab (LB)	RS	-	5 × 10^3^	[23]
Ab-Ab/HRP (SW)	ChA	Physical	5 × 10^3^	[8]
		Covalent	20	
Ab-PEI	QCM	10^5^-5 × 10^8^	6 × 10^4^	[24]
Ab/SWNT/HRP	Colorimetry	-	10^3^	[7]
PZT/Au	QCM	Label-free	5 × 10^3^	[25]
GED-SAM-AuNP	EIS	1 × 10^2^-1 × 10^5^	1 × 10^2^	[26]
Ab-peroxidase	OGC	-	1.3 × 10^3^	[27]
**Ab-As-grown DWNT (Label-free)**	**ChA**	**Label-free 10**^**2**^**-10**^**7**^	**8.9**	**This work**

*Abbreviations: *Ab* antibody, *Au* gold, *Cu* copper, *SW* sandwich, *MgB* Magnetic beads, *LB* Langmuir-Blodgett, *MET*, Magneto elastic sensor, *HRP* horse radish peroxidase, *PEI* polyethyleneimine, *PZT-Au* lead titanate zirconate/gold-coated glass cantilevers, *GED-SAM-AuNP* grafted ethylene diamine & self-assembly gold nanoparticles, *AP* alkakine phosphatase, *ASV* anodic stripping voltammetry, *AWD* Acoustic wave device measurement, *RS* Resonance frequency, *ChA* Chronoamperometry, *QCM* Quartz crystal microbalance, *EIS* Electrochemical impedance spectroscopy, *OGC*, Optical grating coupler.

## Conclusions

It is concluded that the potential of 100 mV *vs.* Ag/AgCl provides a very well-defined oxidation peak of the cells at the DW label immunosensor. The anti-Salmonella concentration of 10 μg/mL exhibited the highest signal response. The detection range at the DW immunosensor was 10^2^ to 10^7^ CFU/mL, which is much wider than the commercial ELISA detection range of 10^3^ to 10^5^ CFU/mL. The electrode showed a high specificity to *S. typhimurium* because it provided no current response when the measurement was conducted on the electrode containing *E. coli*. The results, therefore, suggest that the label free DW immunosensor is specifically promising for *S. typhimurium* detection.

## Methods

### Carbon Nanotubes

**Figure 8:**
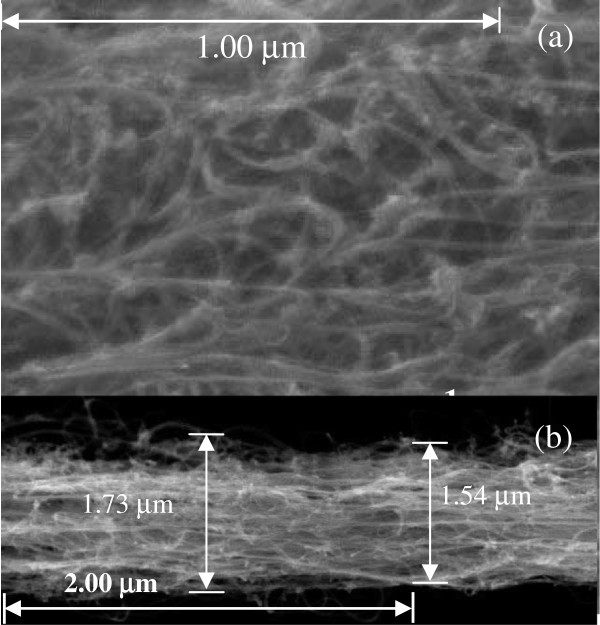
FESEM image of (a) as-grown T-DWNT and (b) a portion of peeled T-DWNT electrode.

CNTs used in the experiment is as-grown-horizontally aligned, thread-like, and double-walled (T-DW). It was obtained from Lijie Ci during his post-doc fellow at Ajayan research group, Rice University, Houston, TX USA with permission from Prof. Pulickel M. Ajayan. The T-DWNT was synthesized and characterized as described in Ci et al. (2002) [27]. The peeled T-DWNT strand diameter was approximately 1–2 μm as shown in Figure 8.

### Chemicals and microorganisms

All chemicals used in the experiment were analytical grade. N-hydroxysuccinimide (NHS), bovine serum albumin (BSA) and 2-morpholineethanesulfonic acid monohydrate (MES) were purchased from Sigma-Aldrich (USA). N-(−3-Dimethylaminopropyl)-N-ethyl-carbodiimide hydrochloride (EDC) was purchased from Fluka (USA). All microbiological media were obtained from Difco (USA). Rabbit anti-*Salmonella* spp. monoclonal antibody was purchased from Biodesign International (USA). Glycine, all buffer reagents, and other chemicals were obtained from Merck (USA). Milli-Q water and double-distilled water were used throughout the experiment. *Salmonella typhimurium* and *Escherichia coli* were obtained from BioSensor Lab, King Mongkut’s University of Technology Thonburi, Bangkok, Thailand.

### Bacterial suspension preparation

The active *S. typhimurium* cells were grown in nutrient broth (NB) and incubated at 30 C and at 150 rpm until an approximately cell number of 10^9^ cells/mL was obtained. The cell suspension was then harvested, centrifuged, washed with phosphate buffer saline (PBS) solution (0.10 M pH 7.4), and re-suspended with the same volume of the original solution to obtain the required cell concentrations for the measurement.

### Optimization of the anti-*Salmonella* spp antibody immunoassay

#### *Antibody immobilization and microbial incorporation*

A CNT strand was peeled out from the CNT forest and the electrode fabrication was performed as stated in the literature [13,14]. The anti-*Salmonella* monoclonal antibody (MAb) was covalently immobilized onto the T-DW electrode by using the EDC crosslink method with some modifications. Briefly, 10 μL of a mixture of 400 mM EDC and 100 mM NHS in MES buffer solution (0.1 M, pH 6.0) was dropped onto the T-DWNT electrode surface and incubated for 1 hr at room temperature. Then, the electrode was washed with PBS buffer solution (0.1 M, pH 7.2) three times and dried at room temperature. Next, 10 μL of the MAb solution (10 μg/mL in deionized water) was dropped to cover the whole area of the electrode, and then it was incubated for another 2 hrs at room temperature. The electrode was then passed three times through washing steps using first a PBS (0.1 M, pH 7.2) containing 0.05% (v/v), and then a Tween 20 (PBS-T), followed by deionized water. Then, 10 μL of phosphate buffer saline (PBS) solution (0.1 M, pH 7.2) containing 2% BSA (w/v) was dropped onto the electrode to block the area without the MAb attachment and the same washing steps were conducted after 30 min of incubation at room temperature. The MAb-DW electrode was stored at 4 C in a humid condition until it was used. For the microbial detection, the bacterial cell suspension was dropped on to the electrode, and it was incubated at room temperature for 2 hrs before being put through the washing steps. The electrode was then used for electrochemical characterization.

#### *Electrochemical characterization of the MAb-DW electrode*

Cyclic voltammetry (CV) was employed in a standard three-electrode system. with the MAb-DW electrode, Ag/AgCl, and Pt disk as working, reference and counter electrodes, respectively. The experiment was conducted on PGSTAT12 by using the GPES software acquisition data. This was done to characterize the electrochemical properties of the MAb-DW electrode. The potential was cycled from −1.0 to 1.0 V *vs.* Ag/AgCl at a scan rate of 20 mV/sec in citrate phosphate buffer (0.05 M, pH 5.5) working solution at a fixed MAb concentration (10 μg/mL in deionized water). This was performed in order to obtain an optimum potential range for further amperometric sensing investigations. The CV experiments were sequentially conducted at the same electrode before and after MAb modification, after blocking with BSA (MAg-DW electrode), and also after the *S. typhimurium* cells (10^9^ CFU/mL) were attached to the electrode (cells-MAg-DW electrode).

#### *Immunoassay at the MAb-DW electrode optimization*

**Figure 9:**
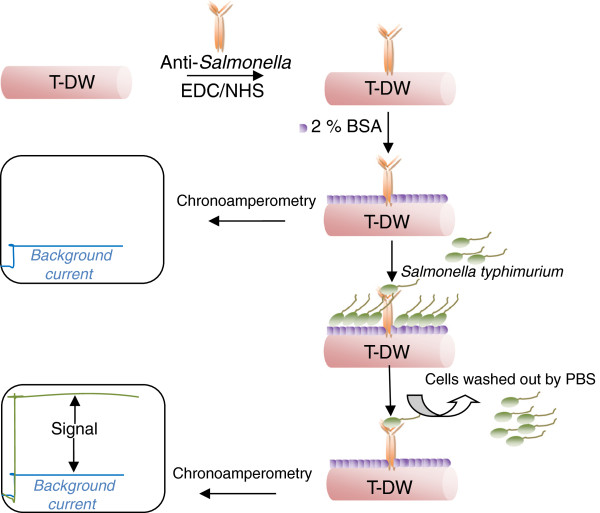
**Schematic of the electrode platform for *S. typhimurium *assay.**

Figure 9 shows the schematic of the electrode platform for *S. typhimurium* assay and how the signal was obtained.

#### *Working potential optimization*

Chronoamperometry (CA) was conducted at the MAb-DW electrode, without the cells incorporated, at a fixed concentration of MAb 10 μg/mL with various working potentials obtained from the CV measurements. These measurements were used to obtain the optimum working potential for cell detection. In the CA measurement, the potential was fixed at 0 V for 100 sec, then stepped to the working potential.for another 250 sec, in which the current response was observed.

#### *MAb concentration optimization*

The signal at the electrode assembled with various MAb concentrations (10, 25, 50 and 100 μg/mL) with and without the attached *S. typhimurium* cells (10^9^ CFU/mL) was observed at the electrode in the citrate phosphate buffer (0.05 M, pH 5.5) working solution. The MAb concentration provided the higher signal and was subsequently further used for sensitivity testing of *S. typhimurium* cells.

### The MAb-DW electrode sensitivity testing

Different *Salmonella* cell concentrations (10-10^9^ CFU/mL) were immobilized on to the MAb-DW electrode and the CA measurements were conducted to obtain the detection range at the electrode. Non-specific adsorption of the Salmonella cells on the BSA-incorporated DWNT electrode was also tested. *Escherichia coli* (10^7^ CFU/mL) was also used for specificity testing at the electrode.

## Competing interests

The authors declare that they have no competing interests.

## Authors’ contributions

NP: carried out the experimental work, participated in data collection and analysis, and was involved in drafting and revising the manuscript. WE: participated in advising and approving the final version of the manuscript to be published. All authors read and approved the final manuscript.
